# Supplementary material for: Prevalence of anemia among Saudi patients with solid cancers at diagnosis in King Faisal Hospital, Taif Province, Kingdom of Saudi Arabia
Source: PLoS One. 2021 Jan 28;16(1):e0246202. doi: 10.1371/journal.pone.0246202 (PMC7842985; doi:10.1371/journal.pone.0246202)
Supplement: Attachment — Submitted filename: Comments to the author.docx [file pone.0246202.s002.docx]

**Comments to the author,**

**Abstract section**,

Line 7- change is to "was". In your objective... newly diagnosed... Does this indicate your study to be prospective?

Line 12- the term incidence preferably good to be replaced by prevalence

Line 15- Add range of your study participants to the age groups

Line 20- P-value in the absence of OR and/or Chi square does not have helpful meaning. So, here the analysis and result should be re-analyzed with the help of OR, chi square and 95% C.I to see association between the variables and to determine clinical significance.

**Introduction section**,

Line 38-40: the term" erythrocytosis" seems not necessary in this paper as it is not relevant to the topic of study

Line 46: replace adversarial with the word" adverse"

**Under Materials and methods section,**

What is your actual study population? Is your study participants patient or medical records? How do you process your ethical assurance?

Line 69: You did not cite for referencing cut off value for anemia and again it lacks in reference section

How did you get 320 of your sample size? Sample size calculation is not stated. What type of sampling technique did you use to collect your sample?

What is your study design? Is it retrospective or prospective?

**In your analysis part**,

Line 84: Even if you mentioned some analytical parameters like Chi square and ANOVA test, it lacks applicability in your findings. What is the value of using ANOVA test?

**In result section**,

Line 94: 56-74 years

Line 106: What is the operational definition base line? Is it consistent with your objective?

Generally, it is not well stated. Only descriptive statistics were used. Majorly, OR and or Chi-square were missed; re-analysis should be operated especially in Table 1 &2. Even the existing p-value is very vague. I t is not clear how p-value is analyzed. For each variable, there should be p-value except constant variable. Again in Table 1 with gender variable, p-value=0.00. By any means, p-value should no be zero. So, re-construct these tables using OR and/or Chi-square, 95%C.I and/or p-value for each independent variable except the constant one.

**Discussion part**,

Line 125 & 131: The same reference is cited [20]; in fact it is not the same. Check your citation and references, too for correction.

Line 125-127; 136-138 & 151-153: Unless there is reference for your possible reason of the differences between your findings and others, it seems personal judgment, which is not scientifically acceptable.

Line 164: It is not clear. Does the statement belong to your finding?

**In reference section**,

Ref No 20 & 22 are the same. Could you address the issue?
